# Supplementary material for: Structural basis for the bi-specificity of USP25 and USP28 inhibitors
Source: EMBO Rep. 2024 May 30;25(7):2950–73. doi: 10.1038/s44319-024-00167-w (PMC11239673; doi:10.1038/s44319-024-00167-w)
Supplement: Supplementary file 1 — Appendix [file 44319_2024_167_MOESM1_ESM.pdf]

Appendix

Structural basis for the bi-specificity of USP25 and USP28 inhibitors

Jonathan Vincent Patzke, Florian Sauer, Radhika Karal Nair, Erik Endres, Ewgenij Proschak, Victor Hernandez-Olmos, Christoph Sotriffer and Caroline Kisker

Content	Page
Appendix Figure S1:<br>Inhibitor electron density<br>Catalytic activity of USP28Δtip variants<br>Ligplot representation of the USP28 inhibitor binding sites	2
Appendix Figure S2:<br>Structural stability of the cat Δtip variants	3

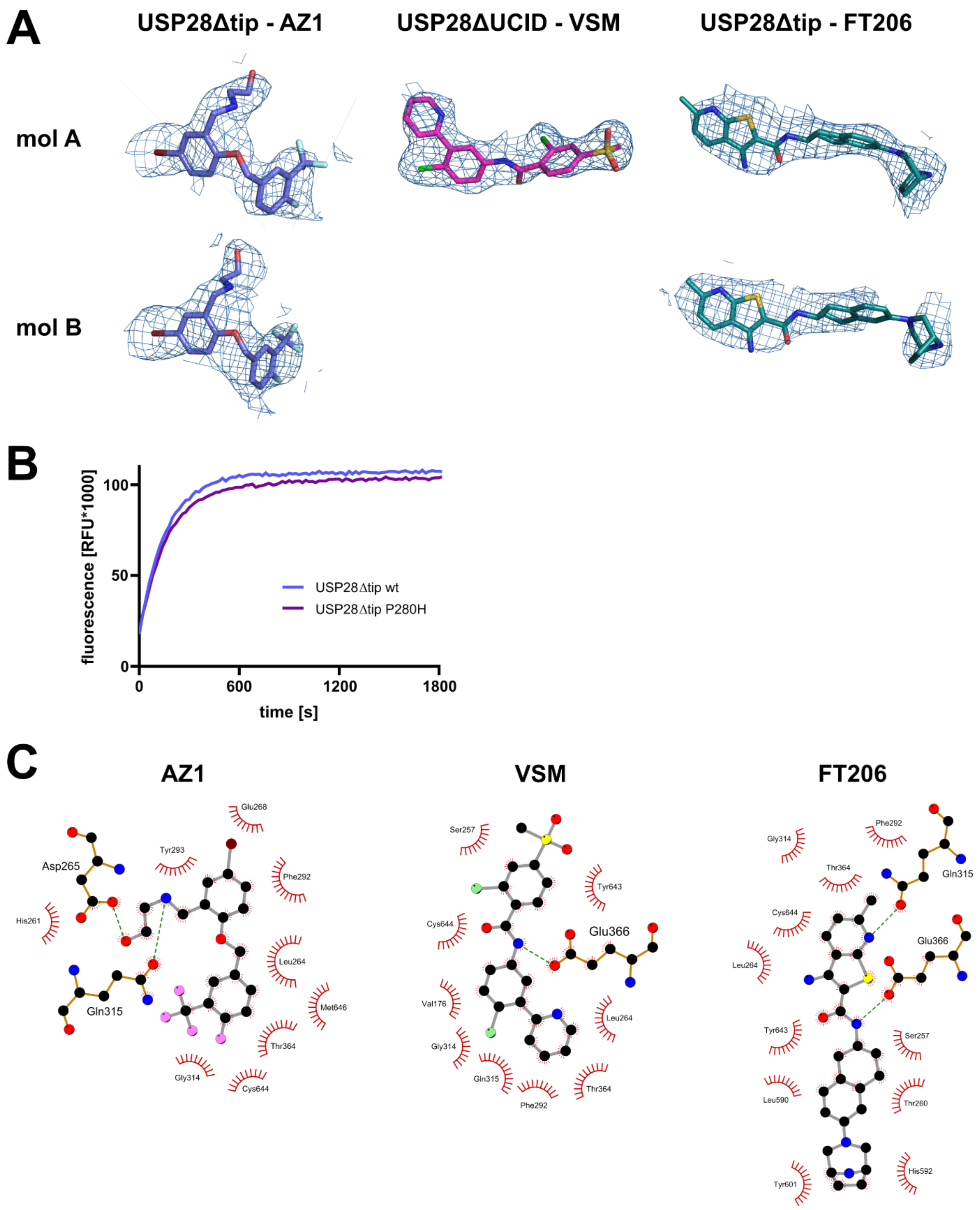

Appendix Figure S1:

A) Inhibitor electron density. 2mFo-DFc simulated annealing (SA) composite omit maps (1 σ level) of AZ1, VSM and FT206 bound to USP28Δtip (molecules A/B) or USP28ΔUCID (molecule A). Electron density for the second FT206 molecule (molecule B) is not present for the complete inhibitor.

B) Catalytic activity of USP28Δtip variants: Representative Ub-Rh110 cleavage assay of USP28Δtip variants (wt (blue) and P280H (purple)). The fluorescence signal [RFU*10^3] is plotted against the time [s].

C) Ligplot representation of the USP28 inhibitor binding sites. Ligplot representation of the USP28 binding sites for AZ1 (left), VSM (center) and FT206 (right).

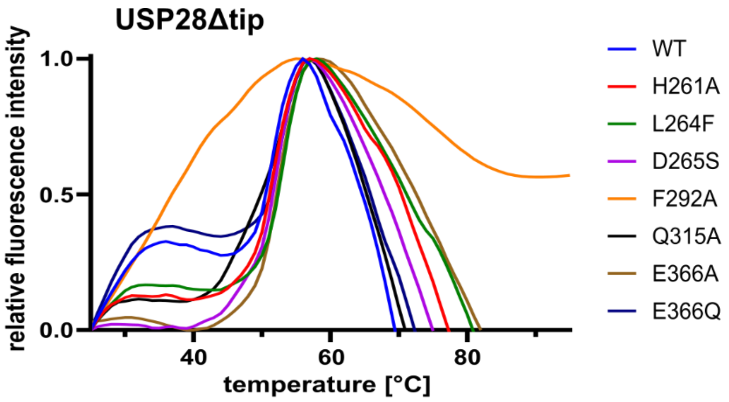

USP28 constructs	Tm [°C]
WT	50.8 ± 2.4
H261A	52.0 ± 0.0
L264F	53.8 ± 0.4
D265S	53.0 ± 0.0
F292A	38.2 ± 2.3 ****
Q315A	53.0 ± 0.0
E366A	52.0 ± 0.0
E366Q	52.5 ± 0.5

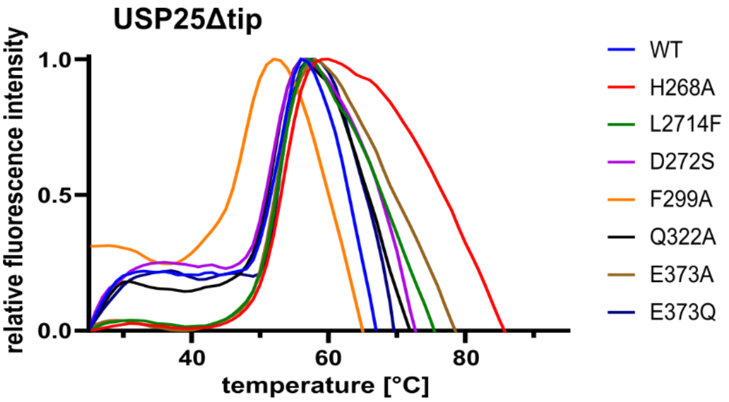

USP25 constructs	Tm [°C]
WT	52.4 ± 0.8
H268A	52.8 ± 0.8
L271F	53.3 ± 0.5
D272S	49.4 ± 3.8
F299A	47.0 ± 0.0 ***
Q322A	52.7 ± 0.5
E373A	52.0 ± 0.0
E373Q	52.2 ± 0.4

Appendix Figure S2: Structural stability of the cat Δtip variants

Melting curves of USP28 (top) or USP25 (bottom panel) Δtip variants measured by differential scanning fluorimetry. One representative experiment is shown for each variant of both proteins. Fluorescence intensity is plotted against the temperature. Melting points were calculated and the melting temperatures [°C] are given in the Table as averages of n ≥ 5 independent experiments ± SD. All the statistics show the results of unpaired Student's t tests, with p-values (* ≤ 0.05; ** ≤ 0.01; *** ≤ 0.001; **** ≤ 0.0001).
