# Supplementary material for: Structural basis for the bi-specificity of USP25 and USP28 inhibitors
Source: EMBO Rep. 2024 May 30;25(7):2950–73. doi: 10.1038/s44319-024-00167-w (PMC11239673; doi:10.1038/s44319-024-00167-w)
Supplement: Supplementary file 10 — Expanded View Figures [file 44319_2024_167_MOESM10_ESM.pdf]

Expanded View Figures

Figure EV1. USP28 and USP7-inhibitor complex structures (corresponds to main Fig. 1).

(**A**) USP28-inhibitor-bound structures. Superposition of the complex structures of USP28 with AZ1 (blue), VSM (magenta) and FT206 (teal) and closeup view on the inhibitor-binding site. Helices α5 are highlighted in the same colors as the inhibitors. (**B**) USP7-inhibitor complex structures. Superposition of structures of USP7 apo (PDB 1NB8) (Hu et al, 2002; Data ref: Hu et al, 2003a), Ub-bound (PDB 1NBF) (Hu et al, 2002; Data ref: Hu et al, 2003b) and in complex with the thumb-palm cleft binding inhibitors Cpd2 (PDB 5WHC; bright green) (Di Lello et al, 2017; Data ref: Murray et al, 2017a), GNE6776 (Kategaya et al, 2017; Data ref: Murray et al, 2017b) and the catalytic channel binding inhibitors ALM2 (PDB 5N9R; dark green) (Gavory et al, 2018; Data ref: Harrison et al, 2017) and FT827 (PDB 5NGF; cyan) (Turnbull et al, 2017; Data ref: Krajewski et al, 2017). Helices corresponding to USP28 α4, α5 and α6 are colored in light red (apo), yellow (Ub-bound) or gray (inhibitor-bound), respectively. The thumb-palm cleft, the catalytic channel as well as the catalytic cysteine (C223) of USP7 are marked. The USP7 helix corresponding to the USP28 α5 moves upon Ub-binding towards the catalytic channel. This movement is blocked by the displayed inhibitors binding to the thumb-palm cleft or the catalytic channel. (**C**) Detailed views of the inhibitor-binding sites. AZ1 (left; blue), VSM (center; magenta) and FT206 (right; teal) are shown with the electron density maps of the inhibitors (at 1 σ). Residues of the thumb-palm cleft binding site are shown as sticks. Hydrogen bonds between the inhibitors and USP28 are displayed as dashed lines.

▶

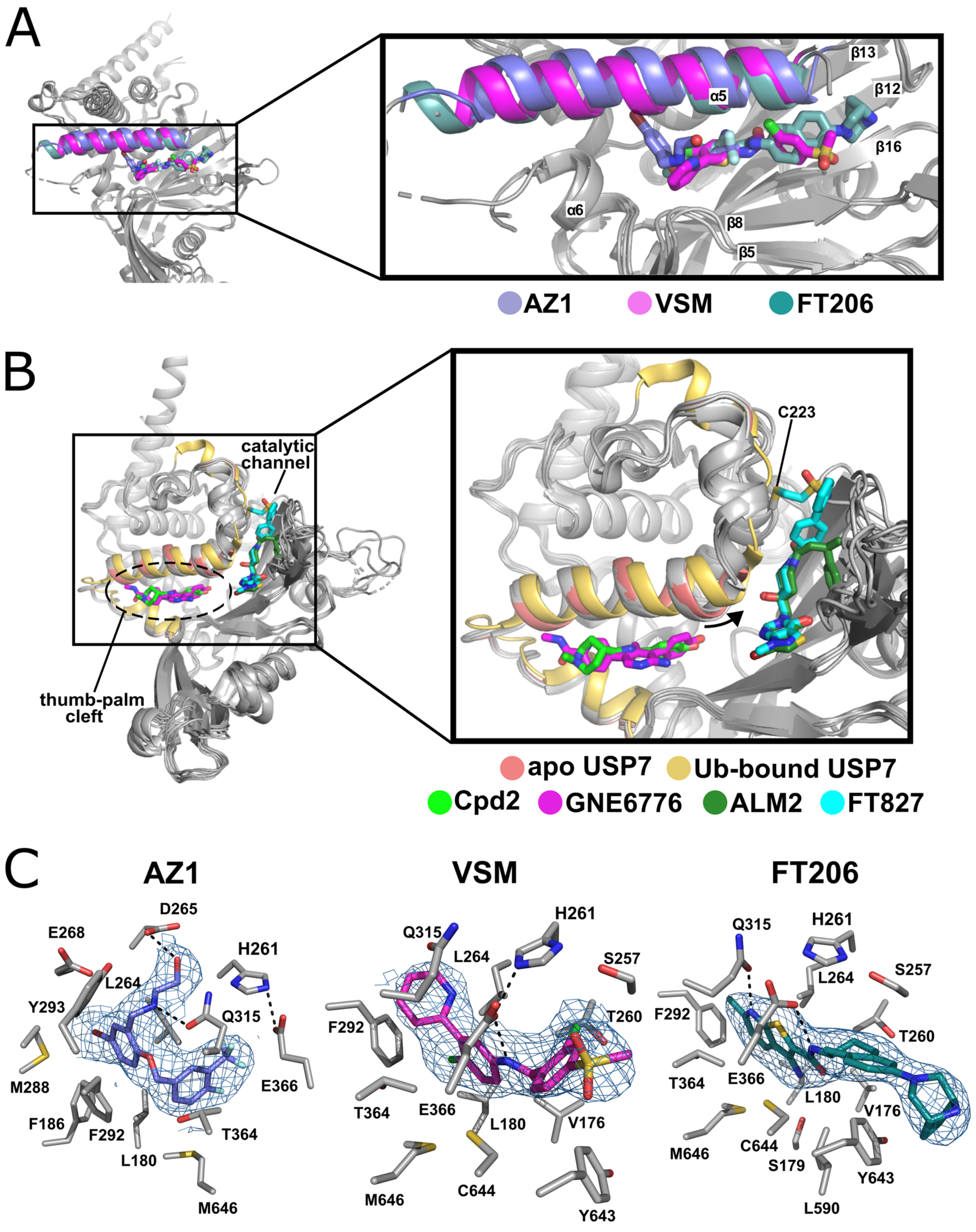
A
β13
β12
α5
β16
α6
β8
β5
AZ1
VSM
FT206
B
catalytic channel
thumb-palm cleft
C223
apo USP7
Ub-bound USP7
Cpd2
GNE6776
ALM2
FT827
C
AZ1
VSM
FT206
D265
E268
H261
L264
Y293
Q315
M288
E366
F186
F292
T364
L180
M646
Q315
H261
L264
S257
F292
T260
T364
E366
L180
V176
C644
M646
Y643
Q315
H261
L264
S257
F292
T260
T364
E366
L180
V176
M646
C644
S179
Y643
L590

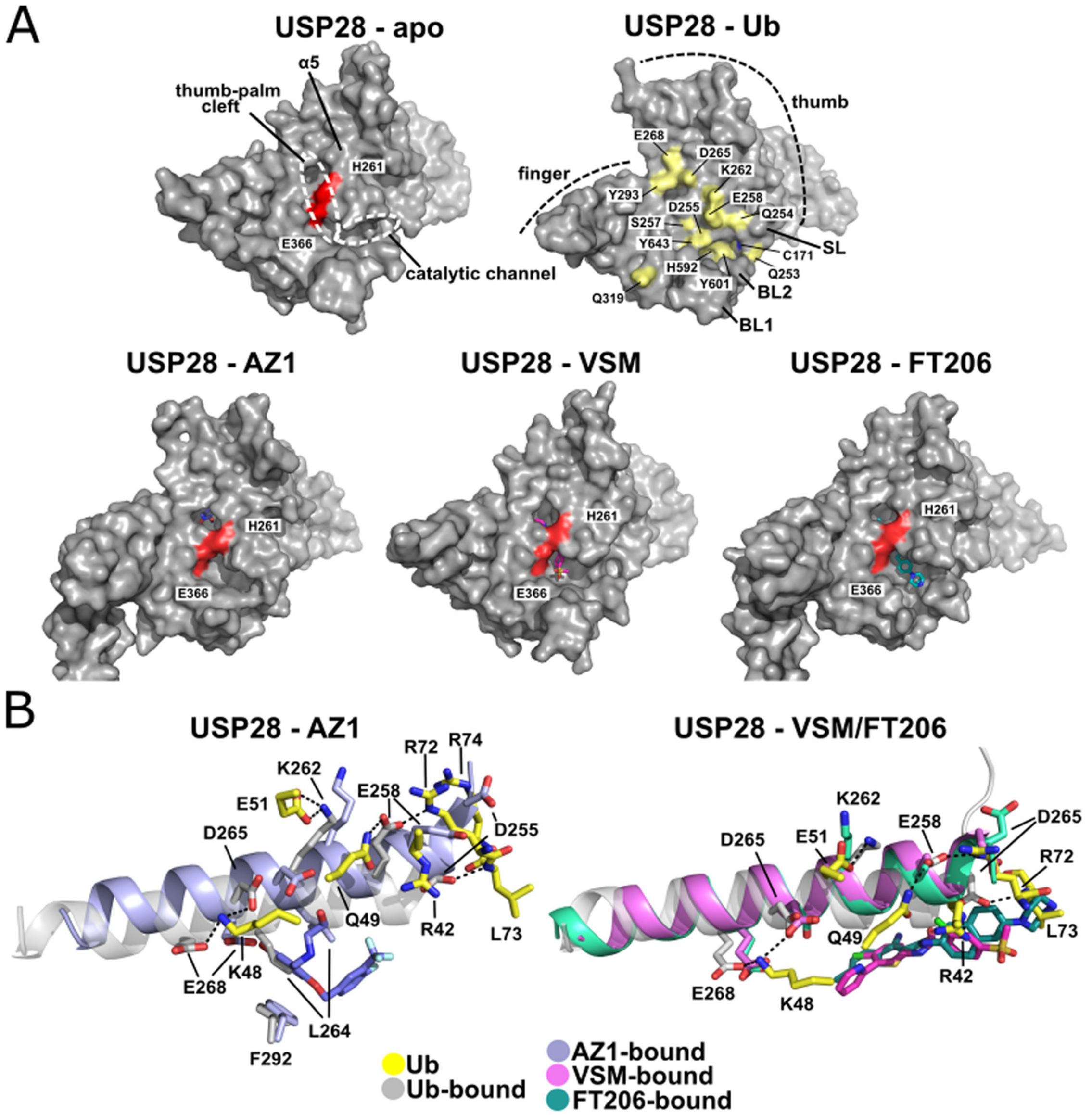

Figure EV2. Inhibitory mechanism (corresponds to main Fig. 2).

(**A**) Comparison of USP28 apo, Ub- and inhibitor-bound states. Top panel: Surface view of the USP28ΔUCID apo (this work) and Ub-bound (PDB 6HEI) (Gersch et al, 2019; Data ref: Gersch and Komander, 2019a) states. Transition from the apo- to the Ub-bound state is accompanied by the reshaping of the S1-site involving closure of the partially open thumb-palm cleft and a widening of the catalytic channel by movement of helix α5 (marked in the apo state). Most residues forming hydrogen bonds and salt bridges with Ub in the S1-site (marked yellow) cluster around the thumb-palm cleft and the catalytic channel. The catalytic cysteine (C171) is highlighted in blue. Bottom panel: Surface view on the inhibitor-bound states. Binding of AZ1, VSM and FT206 in the thumb-palm cleft locks the domain in an apo-like state, where the cleft cannot be closed. Side chains of H261 and E366 which lock the inhibitor-binding sites to the solvent, are highlighted in red. Note that in the apo- and inhibitor-bound states, some mobile elements of the catalytic channel (BL1, SL) are not completely modeled. It therefore appears to be more narrow in the Ub-bound state. (**B**) Closeup view on helix α5 of inhibitor-bound USP28. USP28Δtip P280H—AZ1 (blue, left panel), USP28ΔUCID wt—VSM (pink, right panel) and USP28Δtip wt— FT206 (teal, right panel) superimposed with Ub-bound USP28ΔUCID (PDB 6HEI) (Gersch et al, 2019; Data ref: Gersch and Komander, 2019a). Residues of helix α5 involved in hydrogen bonds and salt-bridge formation with Ub and corresponding positions in the inhibitor-bound state are shown as sticks and hydrogen bonds with Ub as dashed lines.

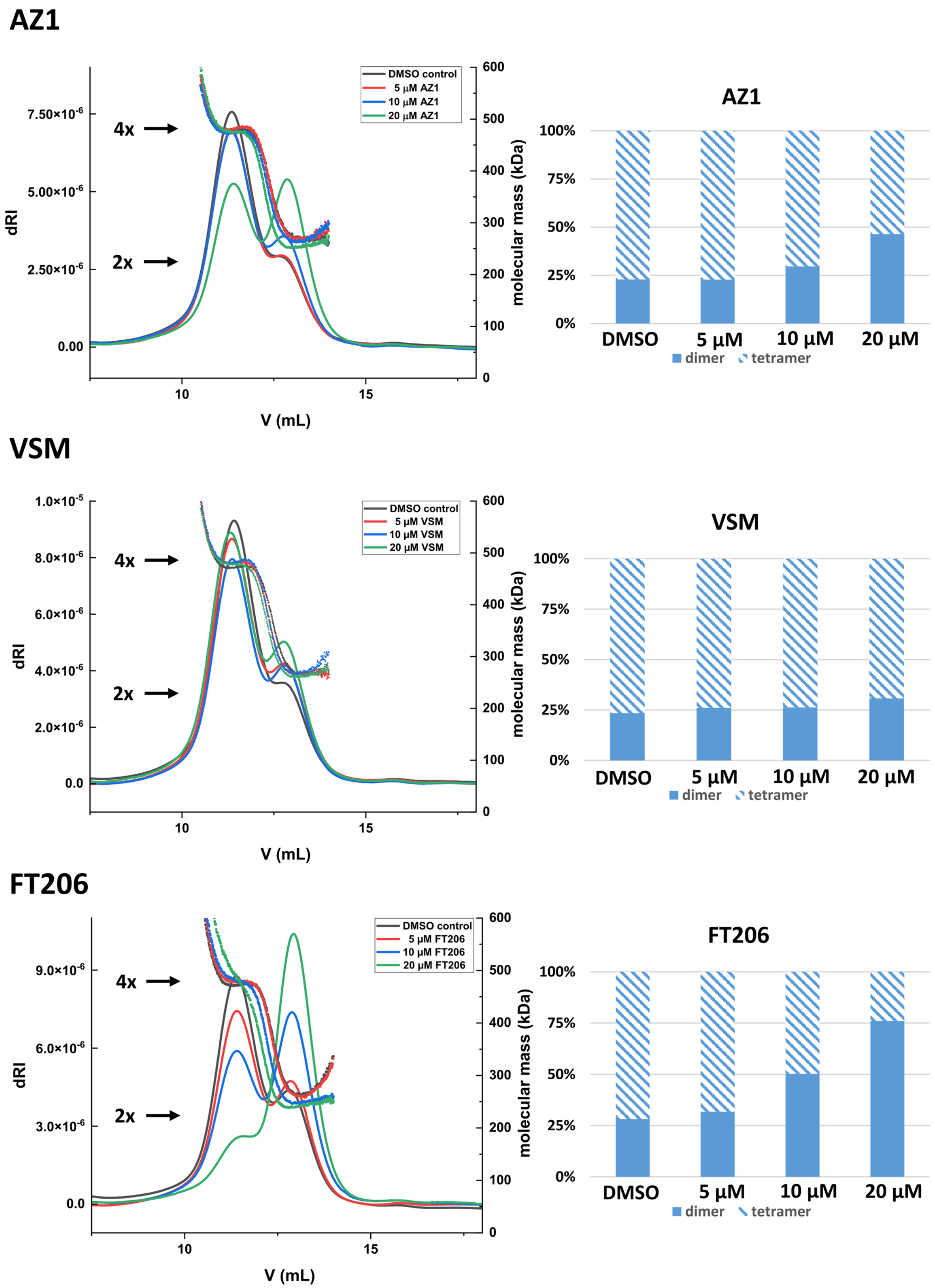

Figure EV3. Inhibitor concentration-dependent dissociation of USP25 tetramers (corresponds to main Fig. 3).

SEC-MALS analysis of 10 µM USP25fl after 45 min incubation with DMSO (black) or 5 µM (red), 10 µM (blue) and 20 µM (green) of AZ1, VSM or FT206 are displayed in the left panels from top to bottom, respectively. Continuous lines represent the protein concentration signal (refractive index, RI). Dots show the molecular mass calculated from RI and light scattering. Fractions of dimers and tetramers calculated from the average molecular mass of the entire peak fraction, each from a single experiment are represented in the bar diagram on the corresponding right panels. Source data are available online for this figure.

 © The Author(s)

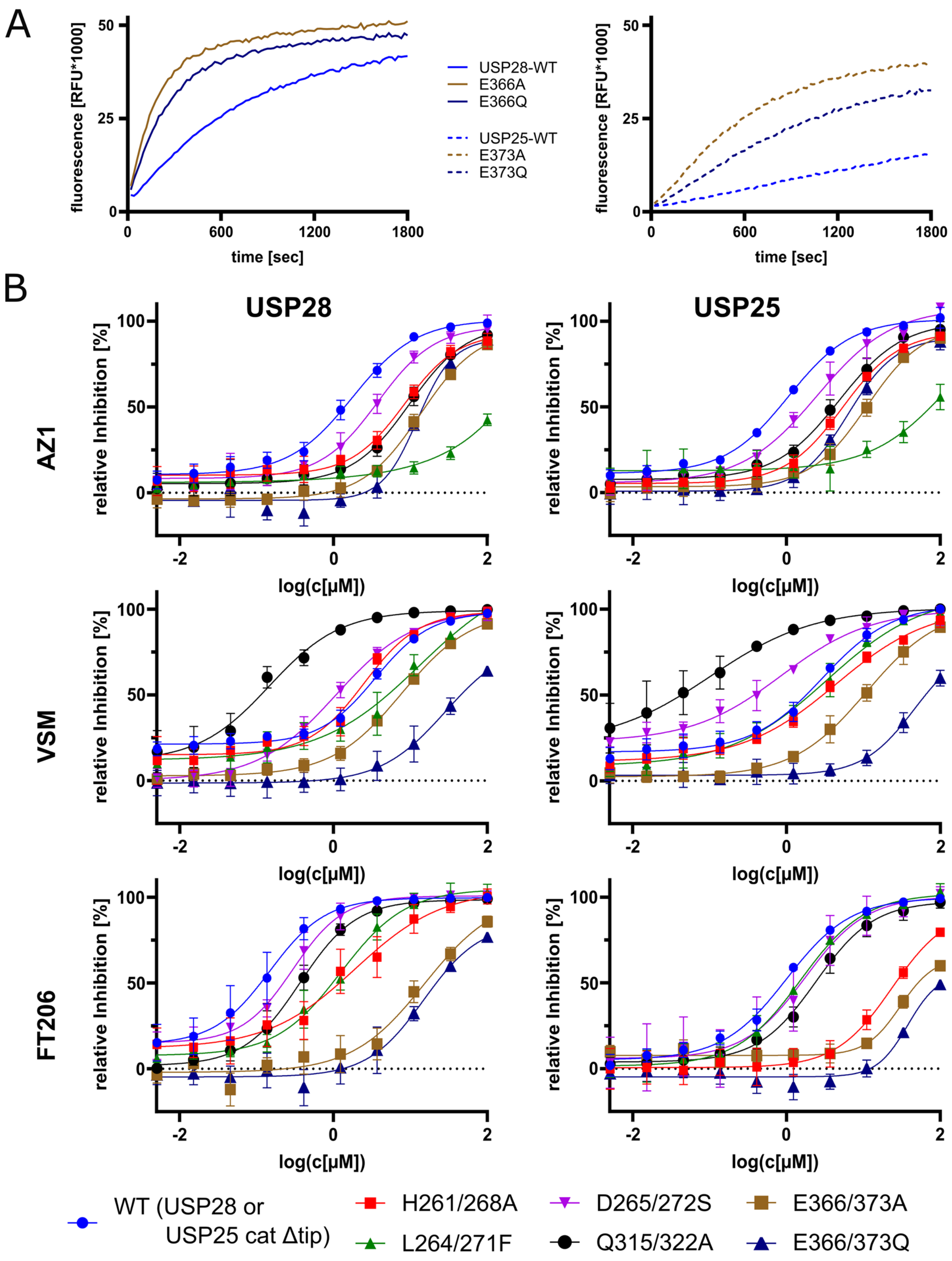

Figure EV4. (corresponds to main Fig. 5).

(**A**) Catalytic activity of wt and hyperactive USP28 and USP25 variants. Representative Ub-Rh110 cleavage assay of USP28Δtip (left panel) or USP25Δtip (right panel) variants (wt (blue), E366A/E373A (brown) and E366Q/E373Q (dark blue). The fluorescence signal (RFU*10^3) is plotted against the time [s]. (**B**) Inhibitory potencies of AZ1, VSM and FT206. Non-linear regression of the dose–response assay for the different USP28 (left) and USP25 (right panels) Δtip variants with AZ1, VSM and FT206, from top to bottom, respectively. Dots represent the mean ± SD ($n \geq 5$, with two biological replicates) at corresponding inhibitor concentrations. The calculated IC_{50} values are depicted in Fig. 4C. Source data are available online for this figure.

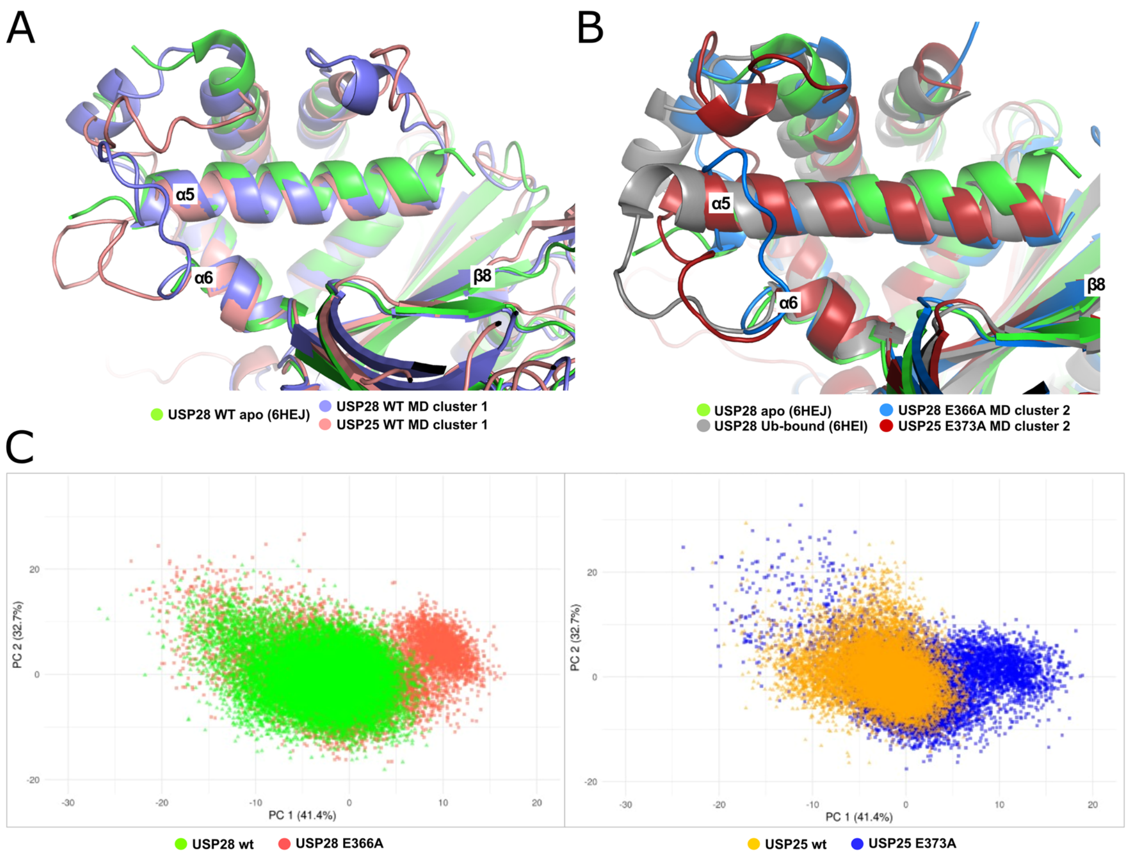

Figure EV5. (corresponds to main Fig. 6).

(**A**) Superposition of the representative snapshots of cluster 1 for USP25ΔUCID (pink) and USP28ΔUCID (blue) with the corresponding crystal structure of apo USP28ΔUCID (PDB 6HEJ; green) (Gersch et al, 2019; Data ref: Gersch and Komander, 2019b). Secondary structure elements containing residues mediating bonds with inhibitor (α5, α6 and β8) are marked. (**B**) Superposition of the crystal structure of apo USP28ΔUCID (PDB 6HEJ; green) (Gersch et al, 2019; Data ref: Gersch and Komander, 2019b) and Ub-bound (PDB 6HEI; gray) (Gersch et al, 2019; Data ref: Gersch and Komander, 2019a) with representative snapshots of cluster 2 for USP28ΔUCID E366A (blue) and USP25ΔUCID E373A (red). Secondary structure elements containing residues mediating bonds with inhibitor (α5, α6, β5 and β8) are marked. (**C**) Two-dimensional principal subspace displaying the differences between wt and E366A/E373A of USP28 (left) and USP25 ΔUCID variants (right panel). For the initial PCA one frame every 100 ps was utilized for the MD trajectories of USP28ΔUCID E366A and the eigenvectors were constructed based on the ten N-terminal backbone atoms of helix α5 and an alignment onto two adjacent β-strands (L363-F370 and Y643-N649). The first two principal components PC1 and PC2 together capture about 74% of the variance within these coordinates (red). All further PCA calculations, both for USP28 wt (green), USP25 wt (yellow) and E373A (blue) were performed utilizing the same alignment and atom selection as described above. The coordinates were expressed in terms of PC1 and PC2 of USP28 E366A to ensure direct comparability between both wt and E366A/E373A as well as USP25 and USP28. Source data are available online for this figure.

 © The Author(s)
